# Efficacy of decompressive craniectomy: A retrospective case series study with 321 patients and an update on controversies

**DOI:** 10.3892/mi.2024.188

**Published:** 2024-08-06

**Authors:** Charalampos Gatos, George Fotakopoulos, Anastasia Tasiou, Grigorios Christodoulidis, Vasiliki Epameinondas Georgakopoulou, Theodosis Spiliotopoulos, Adamantios Kalogeras, Pagona Sklapani, Nikolaos Trakas, Konstantinos Paterakis, Kostas N. Fountas

**Affiliations:** 1 Department of Neurosurgery, General University Hospital of Larissa, 41221 Larissa, Greece; 2 Department of General Surgery, General University Hospital of Larissa, 41221 Larissa, Greece; 3 Department of Pathophysiology, National and Kapodistrian University of Athens, 11527 Athens, Greece; 4 Department of Biochemistry, Sismanogleio Hospital, 15126 Athens, Greece

**Keywords:** decompressive craniectomy, intracranial hypertension, middle cerebral artery ischemic event, Glasgow outcome scale, neurological outcomes

## Abstract

Decompressive craniectomy (DC) is considered a cornerstone in the management of refractory intracranial hypertension. For decades, DC was known as an occasionally lifesaving procedure; however, it was associated with numerous severe complications. The present study is a single-center retrospective case series study on with 321 patients who underwent DC between January, 2010 and December, 2020. All patients were divided into four groups as follows: Group A included patients who suffered from a space-occupying middle cerebral artery (MCA) ischemic event; group B included individuals who developed intracerebral hemorrhage; group C included patients admitted for traumatic brain injury; and group D included patients with other neurosurgical entities that underwent DC, such as subarachnoid hemorrhage, tumors, brain abscess and cerebral ventricular sinus thrombosis events. The present study enrolled a total of 321 patients who underwent DC. Group A included 52 out of the 321 (16.1%) patients, group B included 51 (15.8%) patients, group C included 164 (51.0%) patients, and group D included 54 (16.8%) patients. Of the 321 patients, 235 (73.2%) were males, and the median age was 53.7 years. Multivariate analysis revealed that only the group A parameter was an independent factor associated with a Glasgow outcome scale score >2 during follow-up (P<0.05). On the whole, the results of the present study suggest that among patients who underwent DC with different neurological entities, those who had experienced MCA events had more favorable outcomes.

## Introduction

Decompressive craniectomy (DC) is considered a cornerstone in managing refractory intracranial hypertension (1,2). Proposals suggest that the use of DC can significantly improve the increased intracranial pressure (ICP) values by facilitating the expansion of the edematous cerebral hemispheres (1,3). Furthermore, some researchers have argued that DC disrupts the vicious cycle of intracranial hypertension by reducing cerebral perfusion pressure (1,4).

In late 1890, Kocher introduced the concept of opening-box decompression, which involves removing a variable amount of calvaria (1). In 1901, Kocher proposed opening the skull to relieve increased intracranial pressure, and Cushing (5) performed a subtemporal DC to treat moribund edema due to an intracranial neoplastic disorder. Miyazaki (6) initially described the concept of large cranial and dural decompression, along with the removal of any underlying space-occupying lesions in 1971, while Kjellberg and Prieto (7) refined this surgical technique in 1971.

For decades, DC was known as an occasionally lifesaving procedure, associated, however, with numerous serious complications (8,9). Therefore, the majority of neurosurgeons were not very eager to incorporate DC into the trauma neurosurgical armamentarium. Characteristically, in 1968, Clark (10), stated that the only reason for reporting his experience of performing DCs in patients with of severe traumatic brain injury (STBI) was to warn other neurosurgeons to avoid performing similar surgery. Advancements being made in neuroimaging, prehospital care, and neurointensive care and rehabilitation services have led to a reconsideration of DC as the final treatment option for refractory intracranial hypertension (1,11,12).

In 1999, Guerra *et al* (13) presented a satisfactory outcome in 56% of cases with STB treated with DC, leading to the re-introduction of DC. Since then, the literature has thoroughly discussed the use of DC in STBI cases, leading to numerous retrospective series and randomized controlled trial (RCTs), with controversial results (14,15). Researchers have evaluated this promising new intervention in various neurosurgical emergencies, including stroke, malignant middle cerebral artery (MCA) infarcts (MMCA), acute subarachnoid hemorrhage (SAH), tumor cases, large intracerebral hemorrhage (ICH), cerebral vein thrombosis (CVT) and severe intracranial infections (1). In the literature, there are an ample amount of encouraging studies with extended indications (1,3,4).

According to Professor Servadei, DC was considered a panacea (a cure for everything) for various pathological entities (11). However, DC is an aggressive amputative procedure that is associated with high mortality rates, higher morbidity rates and various types of complications (seizure, subdural hygroma, hydrocephalus, local infection, bone graft resorption and refractory cerebral edema following cranioplasty) (16-18). Conversely, scholars have extensively discussed and assessed the complications associated with DC (19,20). The proportion of cases that may have functional outcomes is relatively small, and that will be feasible after a long-term hospital stay and long-term, high-quality rehabilitation services (21,22).

Every active neurosurgeon is familiar with the procedure in everyday practice; however, the ideal candidate who would benefit from this aggressive amputation procedure is still under investigation and surrounded by controversy. Therefore, it remains debatable whether DC is a panacea or whether it is merely an avenue for other possibilities.

The present study is based on the authors working experience with DCs. This was a single-center retrospective case-series study on 321 consecutive patients who underwent DC. The aim of the present study was to present the authors' experience in dealing with DCs in a single-center retrospective case-series study that included a number of different pathologies, each with a different pathophysiology, clinical course and management, apart from DC and rehabilitation. In addition, under the prisma of new and old controversies, the present study aimed to address which specific patient may benefit from this amputating procedure, as it is considered that the abundant use of DC is not optimal.

## Materials and methods

### Study design and population

The present study was a single-center retrospective study on DC cases. The study included patients that underwent DC in different neurosurgical entities at the authors' local institution (University Hospital of Larissa, Larissa, Greece) between January, 2010 and December, 2020. In total, 321 patients that underwent DC were analyzed in the present study. Data on the age of the patients, sex, a history of anticoagulant use, diabetes and hypertension, site and size of DC external ventricular drain (EVD) placement, post-surgical cases with an ICP ≥20 mmHg, hospital stay and intensive care unit (ICU) stay, Glasgow outcome scale (GOS) and mortality were collected. Patients with an age >75 years were not included (as the comorbidities following craniectomy are increased). The sex as inclusion criteria was not affected our study. All patients were divided into four groups as follows: Group A included patients who suffered from a space-occupying MCA ischemic event; group B included patients who developed ICH; group C included patients admitted for TBI; and group D included patients with other neurosurgical entities that underwent DC, such as SAH, tumors, brain abscess and cerebral ventricular sinus thrombosis (CVST) events. These groups were recognized based on the following demographic, clinical and radiographic data that were reclaimed from the medical archives when available: Age, sex, a history of anticoagulant use, diabetes and hypertension, site and size of DC, EVD placement, post-surgical cases with an ICP ≥20 mmHg, hospital stay and ICU stay, GOS and mortality (Table I). The exclusion criteria were patients that succumbed the first 24 h and cases that were transferred to the ICU in other facilities and thus, lost from the follow-up. All participants had a follow-up period of 1 to 12 years from the day of discharge from the hospital. The primary outcome was defined as a GOS score >2, and the secondary outcomes were the following: Post-surgical cases with an ICP ≥20 mmHg, hospital stay, ICU stay and mortality.

### Surgical technique

The literature reports various types of decompression. DC is unanimously characterized as the typical fronto-temporo-parietal craniectomy/hemicraniectomy that consists of theoretically extensive bone resection, exposing practically the whole underlying cerebral hemisphere (23,24).

The key elements of the procedure included C-spine precautions during positioning. The extended reverse question mark skin incision begins at 1 cm in front of the tragus, extends above and behind the ipsilateral ear, then curves forward 2 cm laterally from the midline, ending just behind the hairline. The decompression extends from the floor of the middle cranial fossa to the zygomatic arch, preserving the superficial temporal artery and the branches of the facial nerve.

The underlying brain edema causes the wound to close in anatomic layers, avoiding tension in the skin margins, and reapproximating the temporalis muscle with a few sutures.

### Statistical analysis

Statistical analyses was performed using the Statistical Package for the Social Sciences (SPSS 11; SPSS, Inc.). Fisher's exact test was used to compare the categorial variables between the groups, and the Mann-Whitney U test for the comparison of continuous data. Data between multiple groups was analyzed using the Kruskal-Wallis test and Steel-Dwass test. To determine the independent contribution of risk factors (explanatory variables) that were statistically significant in the univariable analysis, such as group A (MCA), size of craniectomy-extended (≥120 cm^2^, EVD, post-surgical ICP ≥20 mmHg, hospital stay and mortality, to the development of the GOS (response variable), multivariable analysis was performed. Thus, the risk of an outcome (GOS) may be modified by other risk variables or by their interactions, and these effects can be assessed by multivariable analysis. Linear regression was used with continuous outcomes, while logistic regression was used with binary outcomes. Proportional hazards (Cox) regression analysis was used when the outcome was the elapsed time to an event. A P-value <0.05 was considered to indicate a statistically significant difference. The overall survival time was estimated using Kaplan-Meier analysis and the log-rank test was used to compare the survival curves between groups (group A, 52 patients; group B, 51 patients; group C, 164 patients; group D, 54 patients) for a 1-year observation period.

## Results

**Figure 1:**
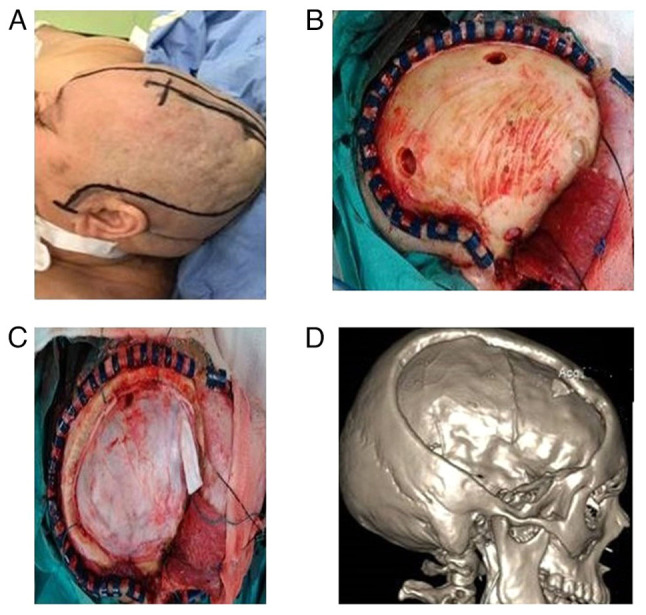
Typical fronto-temporoparietal craniectomy (female patient, 63 years of age, who underwent craniectomy after intracranial tumor removal and extending edema). (A) Skin incision; (B) the burr holes; (C) the underlying dura; (D) 3D craniectomy.

**Figure 2:**
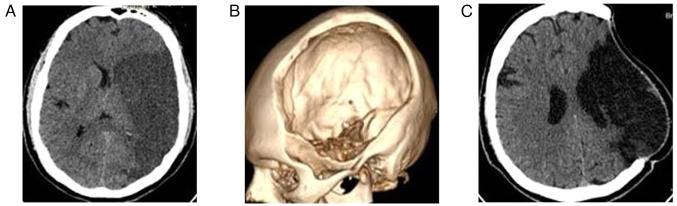
Fronto-temporoparietal craniectomy in a patient with malignant middle cerebral artery stroke (male patient, 48 years of age, with ischemic stroke into the left middle cerebral artery who underwent a left-sides craniectomy and right hemiplegia). (A) Pre-operative CT scan, axial view; (B) 3D craniectomy; (C) postoperative CT scan, axial view.

**Figure 3:**
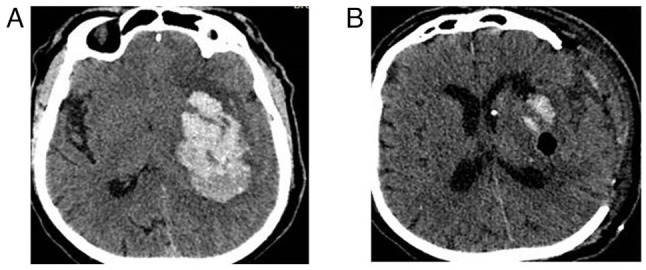
Fronto-temporoparietal craniectomy in a patient with intracerebral hemorrhage (female patient, 52 years of age, with intracerebral hemorrhage into the left side underwent left-side craniectomy and right hemiplegia). (A) Pre-operative CT scan, axial view; (B) post-operative CT scan, axial view.

**Figure 4:**
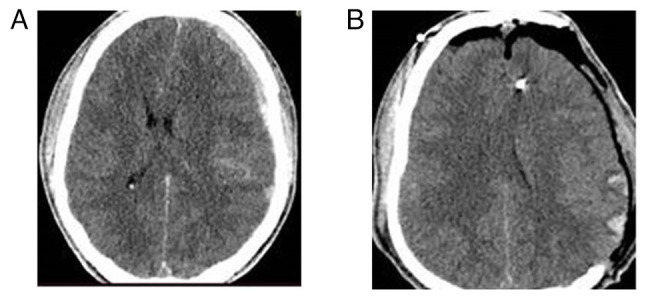
Fronto-temporoparietal craniectomy in a patient with traumatic brain injury (male patient, 18 years of age, with acute subdural hematomas and intra-parenchymal hemorrhages after traumatic brain injury who underwent a left-sided craniectomy and with a favorable outcome). (A) Pre-operative CT scan, axial view; (B) post-operative CT scan, axial view.

**Figure 5:**
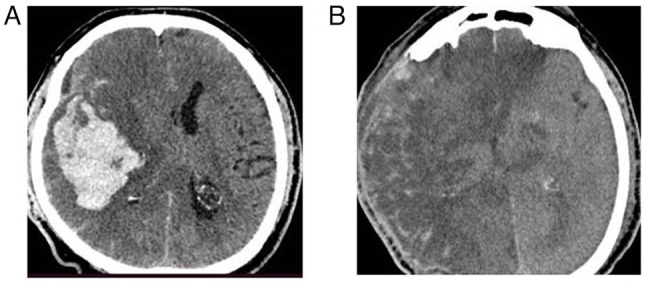
Fronto-temporoparietal craniectomy in a patient with other pathologies (female patient, 64 years of age, who presented with subarachnoid hemorrhage and intracerebral hemorrhage after anterior communicant artery aneurysm rupture and who underwent a right-sided craniectomy with a poor outcome and left hemiplegia). (A) Pre-operative CT scan, axial view; (B) post-operative CT scan, axial view.

The present study enrolled a total of 348 patients who underwent DC (the DC procedure is illustrated in Fig. 1). A total of 27 patients succumbed within the first 24 h or were transferred to the ICU at other facilities and were thus lost from the follow-up. The remaining 321 patients were included in the present study: Group A included 52 out of the 321 (16.1%) patients (Fig. 2); group B included 51 (15.8%) patients (Fig. 3); group C included 164 (51.0%) patients (Fig. 4); and group D included 54 (16.8%) patients (Fig. 5). Of the 321 patients, 235 (73.2%) were males, and the median age was 53.7 years. The patients taking anticoagulant medication were 74 of 321 (23%); those with diabetes were 67 of 321 (20.8%); those with hypertension were 99 of 321 (30.8%); those who underwent a right craniectomy were 121 of 321 (37.6%); those who underwent a left craniectomy were 173 of 321 (53.8%); those who underwent a bilateral craniectomy were 27 (8.4%) and those with EVD placement were 87 of 321 (27%). The baseline characteristics of the patients included in the present study are listed in Table I.

Univariate analysis revealed that there was a statistically significant difference in the patients in group A, in the size of craniectomy, EVD placement, cases with a post-surgical ICP ≥20 mmHg, hospital stay and mortality between the participants who had a GOS score >2 and those who had a GOS score ≤2 (P<0.05, Table II).

**Figure 6:**
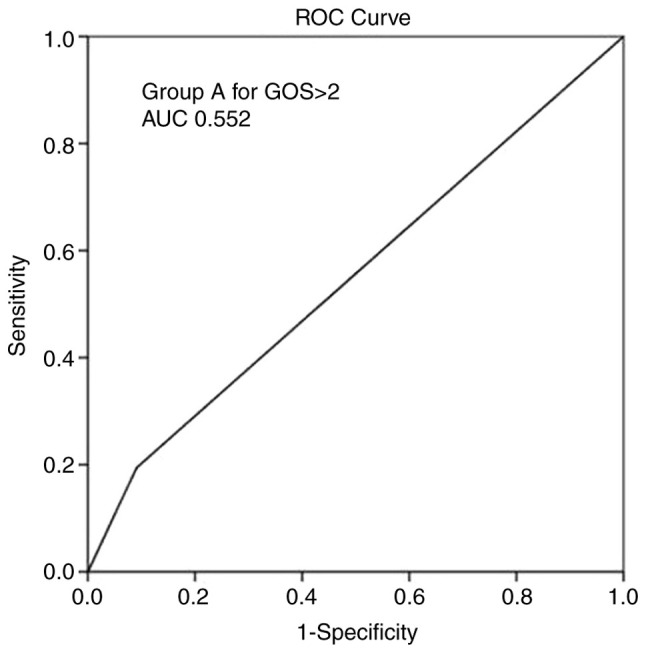
ROC curve for groups (group A), predicting a GOS score >2 during follow-up. AUC, 0.552. AUC, area under the curve; GOS, Glasgow outcome scale; ROC, receiver operative characteristic.

The multivariate analysis (Table III) revealed that the group A (MCA) parameter and mortality were independent factors associated with a GOS score >2 during follow-up (P<0.05). Overall, ROC analysis demonstrated that the group A parameter exhibited the optimal performance to predict a GOS score >2, as evaluated by an area under the curve standard error [AUC (SE)] of [0.552 (0.033)] and (P=0.128) (Table IV and Fig. 6).

**Figure 7:**
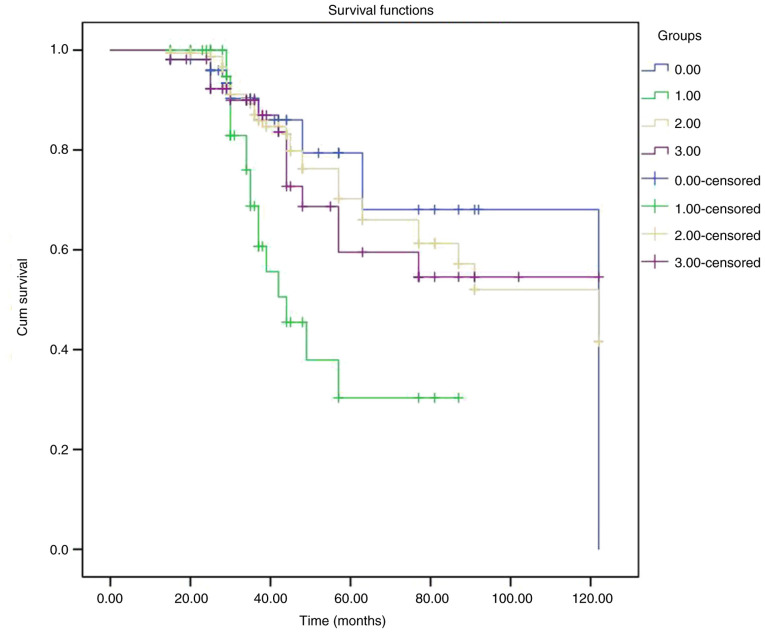
Graph illustrating representative Kaplan-Meier survival curves of the patients who underwent decompressive craniectomy and grouped according to different neurosurgical entities. In the figure, the groups are indicated as follows: Group A (DC, space-occupying middle cerebral artery event), blue color; group B (DC, intracerebral hemorrhage), green color; group C (DC, traumatic brain injury), yellow color; group D (DC, other entities), purple color. DC, decompressive craniectomy.

Kaplan-Meier (Fig. 7) analysis estimated the survival rates of the groups of patients that underwent DC (Table V). The log-rank test revealed a statistically significant difference (P=0.006), indicating the difference between the survival times among the four groups.

## Discussion

The results of the present study suggest that among patients who underwent DC with different neurological entities, those who experienced MCA events had more favorable outcomes with a GOS score >2 and lower mortality rates. Thus, the role of DC in daily clinical practice may be more efficient for these patients.

The literature demonstrates highly variable DC outcome rates in different entities (25). Tagliaferri *et al* (25) retrospectively analyzed 526 consecutive cases treated with DC, reporting poor outcomes in 77% of the cases, which escalated to 93% in patients over 65. For patients aged 18 to 65 years, they stated that the only statistically significant parameters were age, the time of decompression and tge size of the bone flap (25). Goedemans *et al* (26) presented 204 cases of DC, reporting a range of functional outcomes from 91% in CVT cases to 0% in SAH cases with ischemia; 26% of the cases with STBI and 39% of the stroke cases survived independently. Kapapa *et al* (27) assessed 134 cases with various entities treated with DC, and concluded that the outcomes after DC do not differ significantly among patients with different pathologies.

### STBI

The literature extensively assesses the use of DC in patients with STBI (27), using RCTs, numerous retrospective series, consensus and guidelines (12,24,28-31). The results, conclusions and limitations of recently published RCTs are thoroughly considered in the literature (12,24,28-31). The RCTs for STBI have established that DC does indeed lower ICP rates and occasionally, even mortality rates; however, the majority of survivors fail to achieve optimal functional outcomes (28-31). It is important to emphasize that DC does not reverse the effects of the disease, and factors affecting the outcome include age, other concomitant injuries, co-morbidities, chronic medication, substance abuse and the genetic profile of patients. High-quality ICU and rehabilitation services are detrimental in order to achieve the desired outcomes.

There is an increase in the number of survivors in a vegetative state. Thus, there is a belief that DC translates mortality into survival with severe disability and dependency (21,22,31-33). Furthermore, in the literature, there is ample criticism about the vast differences and equalities in the management and outcome of cases with STBI between middle-income countries (MICs) and low-middle-income countries (LMICs) (21,22,31-33). Despite the fact that LMICs and health systems with limited resources report 90% of trauma-related deaths, there is minimal or no participation in RCTs, particularly in the ICU, and the availability of rehabilitation is crucial (21,22,31-33). DC continues to be proposed as a prophylactic measure for the management of STBI, where sophisticated monitoring resources are not feasible (28). Clinical practice has undergone a reassessment due to the lack of solid evidence and the heterogeneity of STBI worldwide. It is recommended that therapy be individualized following a patient-centered discussion about realistic outcome expectations (34).

### MMCA

In the era of thrombolysis and thrombectomy, DC for stroke has earned a distinguished place, particularly in cases of MMCA. DC decreases ICP, improves perfusion and blood flow, and reduces mortality rates. The RCTs of the past two decades have revealed a significant benefit in functional outcomes in all predefined subgroups (35). In the majority of cases, that study reported good outcomes, with a GOS score >2 in 78% of the cases (35). Older patients have a lower margin of benefit (35). There is no evidence that DC affects outcomes when delayed 48 to 92 h following the onset of stroke, but surely before herniation (35). As for dominant infarcts, there is a bias toward worse outcomes related to aphasia; the literature does not support withholding DC based on laterality (36). The decision to perform DC should not be based on ICP values, but if available, post-DC ICP monitoring may be useful (36). Predictors of malignant edema and optimizing provider settings are the key elements of achieving the optimal outcome possible.

### ICH

ICH constitutes a devastating disease with high mortality and morbidity rates (37). Yao *et al* (37), in their systematic review and meta-analysis, stated that DC effectively reduced mortality in cases with ICH, and that DC may improve functional outcomes in certain populations and warrants further verification. In the present study, the results are not so disheartening; however, there is probably a bias, since older individuals and patients with major co-morbidities were not subjected to DC. Additionally, decompression was only applied when the Glasgow coma scale (GCS) score decreases, and certainly not before herniation.

Minimally invasive procedures may be feasible in elective cases. DC with or without hematoma evacuation should be considered as a life-saving approach when neurological deterioration is evolving (38).

### Other neurosurgical entities

There are only retrospective case series available that evaluate other neurosurgical entities treated with DC, including SAH, tumors, brain abscesses and CVST (39). However, the pertinent literature reports a wide variation in clinical outcomes and ill-defined indications for DC in patients (39). RCTs have not provided solid evidence (28-31,35) that DC can ameliorate functional outcomes in the other entities. In the literature, there are highly variable rates of complication mortality morbidity; there is a trend that cases with an SAH have poorer prognoses (39). The results of the present study are positive. Notably, there are case reports assessing DC in entities, such as encephalopathies and diabetic ketoacidosis (40,41).

### Complications

DC has been associated with high mortality rates, mainly due to the severity of the underlying trauma, as well as numerous and occasionally severe complications (20). Researchers have extensively studied and reported peri-operative, early post-operative and late post-operative complications (19,20).

Researchers have identified several factors that predispose to the development of DC-associated complications (20). These include a low GCS score upon admission, the age of the patient, co-morbidity, and the systematic pre-operative anticoagulant administration (20).

### Cranioplasty

A drawback of DC is that a second surgical intervention is required to repair the bone defect (42). There are a number of colleagues who consider that cranioplasty is the normal sequel of DC and that the surgical procedure of CP is an extension of DC. Without any medical contraindications, we should make every effort to perform CP after DC.

Although CP is considered a straightforward procedure, it is well documented that it is associated with considerable complication rates that are widely variable in the literature (42-44).

### DC and survival for different pathologies

When comparing DC for different pathologies, DC for MCA stroke has a trend toward improved outcomes, probably due to the high alert of physicians who address cases to neurosurgical facilities on time and before herniation. The benefit of DC in cases with malignant MCA stroke is well-established in the literature, and studies have documented significant benefits in functional outcomes (35,36). The different groups assessed are highly variable In subsequent pathophysiology and further clinical management, and the categorization in these groups may be arbitrary. The present study reported disheartening results for the ICH cases. ICH constitutes a devastating disease, and the reason to perform DC is lethal intracranial hypertension; without DC the outcome would be a GOS score of 1 or 2.

The present study has certain limitations which should be mentioned. The present study harbors all the limitations of a single-center retrospective case-series study. The heterogeneity of the population, different pathologies with unique pathophysiological sequelae, and diverse treatment modalities other than DC cannot surely fit into one basket. In addition, the different groups assessed are highly variable. In subsequent pathophysiology and further clinical management, the categorization in these groups may be arbitrary; the authors aimed to report their experience in these complex cases and to reveal any trends that may be clinically significant.

In the near future, novel evaluation methods need to be endorsed, as optimizing medical and neurocritical care, optimal provider settings and high-quality rehabilitation services are detrimental to ameliorating the outcome. Determining functional outcomes via the modified Rankin scale, GOS and the GOS extended may not be capable of sufficiently describing all aspects of patients, families, caregivers, surgeons and health system expectations.

Current medicine regularly discusses the major issue of quality of life. Furthermore, the socioeconomic effects create a heavy burden that challenges even sophisticated and plentiful health systems. Environments with limited capabilities drastically exacerbate this issue. These vast distances and differences between HMICs and LMICs constitute a highly controversial topic (21,22,32,33). Subpopulations should also receive special attention due to age-limit comorbidities (34).

Despite the clear postulation that DC cannot cure unsalvageable patients while providing them with a reasonable quality of life, overtreatment remains a contentious issue (8,9).

In conclusion, DC is an aggressive amputation procedure with numerous complications that effectively reduces ICP values and mortality rates. However, the impact on functional outcomes and the quality of life of survivors remains highly controversial, and the current evidence has several limitations. Furthermore, individuals demand high-quality mandatory rehabilitation services and prolong recovery times. It is difficult to withhold DC from a young patient who develops refractory intracranial hypertension and has at least some chance of survival with an accepted disability. One cannot overemphasize the importance of individualized treatment and patient-centered discussions. However, before performing one, the well-documented risks and drastic complications of DC must be seriously considered. In carefully selected cases, these risks may outweigh the expected benefits. Finally, the excessive use of DC could be more optimal.

## Figures and Tables

**Table I:** Baseline demographic characteristics of the patients.

Parameter	All patients, n=321 (100%)	Group A (space-occupying MCA event), n=52 (16.1%)	Group B (ICH), n=51 (15.8%)	Group C (TBI), n=164 (51.0%)	Group D (other), n=54 (16.8%)	P-value
Age, mean ± SD (years)	53.0±19	63.0±13	62.3±12	42.7±19	65.7±10	0.001^b^
Sex (male), n (%)	235 (73.2)	33 (10.2)	34 (10.5)	135 (42.0)	33 (10.2)	0.002^b^
Anticoagulant, n (%)	74 (23.0)	13 (4.0)	15 (4.6)	34 (10.5)	12 (3.7)	0.616
Diabetes, n (%)	67 (20.8)	8 (2.4)	15 (4.6)	29 (9.0)	15 (4.9)	0.123
Hypertension, n (%)	99 (30.8)	11 (3.4)	20 (6.2)	48 (14.9)	20 (6.2)	0.163
Site of craniectomy, n (%)						
Right	121 (37.6)	18 (5.6)	18 (5.6)	63 (19.6)	22 (6.8)	0.568
Left	173 (53.8)	30 (9.3)	32 (9.9)	84 (26.1)	27 (8.4)	
Bilateral	27 (8.4)	4 (1.2)	1 (0.3)	17 (5.2)	5 (1.5)	
Size of craniectomy, n (%)						
Extended (≥120 cm^2^)	231 (71.9)	33 (10.2)	33 (10.2)	129 (40.1)	36 (11.2)	0.055
<120 cm^2^	90 (28.0)	19 (5.9)	18 (5.6)	35 (10.9)	18 (5.6)	
EVD, n (%)	87 (27.0)	12 (3.7)	20 (6.2)	35 (10.9)	20 (6.2)	0.022^a^
Post-surgical ICP, n (%)						
≥20 mmHg	105 (32.7)	11 (3.4)	22 (6.8)	51 (15.8)	21 (6.5)	0.078
<20 mmHg	216 (67.2)	41 (12.7)	29 (9.0)	113 (35.2)	33 (10.2)	
Hospital stay, mean ± SD (days)	42.4±22	40.3±22	35.1±15	43.8±24	46.9±24	0.062
ICU stay, mean ± SD (days)	24.8±16	24.9±17	21.2±13	25.2±16	26.8 ±16	0.390
GOS, mean ± SD	2.8±1	3.4±1	2.5±1	2.8±1	2.7±1	0.003^b^
Mortality, n (%)	72 (22.4)	8 (2.4)	17 (5.2)	33 (10.2)	14 (4.3)	0.119

Group D included patients with other neurosurgical entities that underwent DC, such as subarachnoid hemorrhage, tumors, brain abscess and cerebral ventricular sinus thrombosis events. Data were analyzed using non-parametric tests, namely the Kruskal-Wallis test and the Steel-Dwass test.

^a^P<0.05 and

^b^P<0.01. MCA, middle cerebral artery; ICH, intracerebral hemorrhage; SD, standard deviation; TBI, traumatic brain injury; EVD, external ventricular drain; ICP, intracerebral pressure; ICU, intensive care unit; GOS, Glasgow outcome scale.

**Table II:** Univariate analysis for GOS.

Parameters	GOS score >2, n=211	GOS score ≤2, n=110	P-value
Groups, n (%)			
Group A (space-occupying MCA event)	41 (12.7)	10 (3.1)	0.016^a^
Group B (ICH)	33 (10.2)	18 (5.6)	0.866
Group C (TBI)	100 (31.1)	64 (19.9)	0.066
Group D (Other)	36 (11.2)	18 (5.6)	0.874
Age, mean ± SD (years)	53.7±19	51.7±19	0.396
Sex (male), n (%)	150 (46.7)	85 (26.4)	0.235
Anticoagulant, n (%)	46 (14.3)	28 (8.7)	0.461
Diabetes, n (%)	48 (14.9)	19 (5.9)	0.252
Hypertension, n (%)	68 (21.1)	31 (9.6)	0.456
Site of craniectomy, n (%)			
Right	78 (24.2)	40 (12.4)	0.782
Left	111 (34.5)	62 (19.3)	
Bilateral	19 (5.9)	8 (2.4)	
Size of craniectomy, n (%)			
Extended (≥120 cm^2^)	162 (50.4)	69 (21.4)	0.008^b^
<120 cm^2^	49 (15.2)	41 (12.7)	
EVD, n (%)	48 (14.9)	39 (12.1)	0.015^a^
Post-surgical ICP			
≥20 mmHg	61 (19.0)	44 (13.7)	0.044^a^
<20 mmHg	150 (46.7)	66 (20.5)	
Hospital stay, mean ± SD (days)	41.3±23	44.4±22	0.039^a^
ICU stay, mean ± SD (days)	24.0±15	26.3±16	0.161
Mortality, n (%)	3 (0.9)	69 (21.4)	0.001^b^

Group D included patients with other neurosurgical entities that underwent DC, such as subarachnoid hemorrhage, tumors, brain abscess and cerebral ventricular sinus thrombosis events. Data were analyzed using non-parametric tests, namely Mann Whitney U test.

^a^P<0.05 and

^b^P<0.01. MCA, middle cerebral artery; ICH, intracerebral hemorrhage; SD, standard deviation; TBI, traumatic brain injury; EVD, external ventricular drain; ICP, intracerebral pressure; ICU, intensive care unit; GOS, Glasgow outcome scale.

**Table III:** Multivariate analysis for a GOS score >2.

			95% CI for Exp (B)	
Parameters	P-value	Exp (B)	Lower	Upper
Groups-group A (space-occupying MCA event)	0.030^a^	0.089	0.011	0.219
Size of craniectomy - extended (≥120 cm^2^)	0.657	-0.020	-0.112	0.071
EVD	0.675	0.019	-0.076	0.117
Post-surgical ICP-≥20 mmHg	0.494	0.030	-0.058	0.119
Hospital stay	0.195	-0.052	-0.003	0.001
Mortality	0.001^b^	-0.697	-0.887	-0.700

Data were analyzed using linear regression analysis (regression coefficients: estimates, 95% confidence intervals; model fit; R squared change).

^a^P<0.05 and

^b^P<0.01. Exp (B), the odds ratio value; CI, conﬁdence interval; MCA, middle cerebral artery; ICH, intracerebral hemorrhage; SD, standard deviation; TBI, traumatic brain injury; EVD, external ventricular drain; ICP, intracerebral pressure; ICU, intensive care unit; GOS, Glasgow outcome scale.

**Table IV:** ROC analysis for GOS.

Parameters	Area	Std. error	CI (95%) lower-upper	P-value
Groups-group A (space-occupying MCA event)	0.552	0.033	0.487-0.616	0.128

ROC curve analysis was performed (with standard error and confidence interval) and coordinate points of the ROC curve. Std., standard; CI, confidence interval; MCA, middle cerebral artery; GOS, Glasgow outcome scale (GOS).

**Table V:** Kaplan-Meier analysis for patient survival.

A, Mortality and groups			
Parameters	Estimate (days)	Std. error (days)	CI (95%) lower-upper
Group A (space-occupying MCA event)	97.41	9.4	78.85-115.98
Group B (ICH)	53.69	5.0	43.84-63.55
Group C (TBI)	90.12	4.6	80.92-99.32
Group D (other)	87.28	7.2	73.00-101.56
B, Test of equality of survival distributions in the different groups			
	Chi-squared test value	P-value	
Log-rank (Mantel-Cox)	12.39	0.006^a^	

Kaplan-Meier analysis was performed (mean and median survival). The Chi-square test was used to test the equality of survival distributions in the different groups.

^a^P<0.01. Std., standard; CI, confidence Interval; MCA, middle cerebral artery; EVD, external ventricular drain; ICP, intracerebral pressure.

## Data Availability

The datasets used and/or analyzed during the current study are available from the corresponding author on reasonable request.
